# Mapping the risk of Zika virus infections in pregnant persons and microcephaly in newborns in relation to socioeconomic indicators in Recife, Pernambuco, Brazil: A spatial analysis (2015 to 2021)

**DOI:** 10.1371/journal.pntd.0013240

**Published:** 2025-07-09

**Authors:** Ana Carolyne de Carvalho Lucena Sá, André Luiz Sá de Oliveira, Demócrito de Barros Miranda-Filho, Celina Maria Turchi Martelli, Thalia Velho Barreto de Araújo, Elizabeth B. Brickley, Ricardo Arraes de Alencar Ximenes, Ulisses Ramos Montarroyos

**Affiliations:** 1 Universidade de Pernambuco – UPE, Faculdade de Ciências Médicas, Postgraduate Program in Health Sciences, Recife, Pernambuco, Brazil; 2 Department of Statistics and Geoprocessing, Fundação Oswaldo Cruz – Fiocruz, Aggeu Magalhães Institute, Recife, Pernambuco, Brazil; 3 Department of Public Health, Fundação Oswaldo Cruz – Fiocruz, Aggeu Magalhães Institute, Recife, Pernambuco, Brazil; 4 Department of Social Medicine, Universidade Federal de Pernambuco – UFPE, Recife, Pernambuco, Brazil; 5 Department of Infectious Disease Epidemiology & International Health, London School of Hygiene & Tropical Medicine, London, United Kingdom; 6 Department of Tropical Medicine, Universidade Federal de Pernambuco – UFPE, Recife, Pernambuco, Brazil; University of California Davis School of Veterinary Medicine, UNITED STATES OF AMERICA

## Abstract

**Objective:**

Using spatial analysis techniques, this study investigates the associations between socioeconomic indicators measured at the census tract level and the incidence of Zika virus (ZIKV) infection in pregnant persons and microcephaly in newborns in Recife, Pernambuco, Brazil, from 2015 to 2021.

**Methods:**

In this ecological study, data on cases of ZIKV infections among pregnant persons registered in the Brazilian Information System for Notifiable Diseases (*Sistema de Informação de Agravos de Notificaçao*, SINAN-Zika) and cases of microcephaly among live births registered in the Public Health Event Registration System (*Registro de Eventos em Saúde Pública,* RESP-Microcefalia) and the Live Birth Information System (*Sistema de Informações Sobre Nascidos Vivos,* SINASC) were georeferenced based on residential address and aggregated within census tracts. Spatial autocorrelation was performed using the bivariate global and local Moran’s I indices, which considered the incidence rates of maternal ZIKV infections and microcephaly during the epidemic (2015–2017) and post-epidemic (2018–2021) time periods in relation to each other as well as social, economic, sanitation, and urban infrastructure covariates derived from the 2010 census.

**Results:**

From 2015 to 2021, the city of Recife registered 253 cases (n = 240 in 2015–2017 and n = 13 in 2018–2021) of ZIKV infection in pregnant persons and 147 cases (n = 137 in 2015–2017 and n = 10 in 2018–2021) of microcephaly in newborns. The global bivariate Moran’s I index between the incidences of ZIKV infection in pregnant persons and microcephaly were 0.127 (p = 0.001) in 2015–2017 and 0.074 (p = 0.002) in 2018–2021, indicating a positive spatial correlation, as higher was the ZIKV infection in pregnant persons, higher was the incidence of microcephaly. Furthermore, incidences of maternal ZIKV infection and microcephaly were consistently associated with indicators of greater social vulnerability and economic deprivation at the census tract level.

**Conclusion:**

During the epidemic and post-epidemic periods in Recife, cases of ZIKV in pregnant persons and microcephaly were concentrated in census tracts with relatively higher socioeconomic vulnerability, reinforcing the need for research to inform the development of social protection and environmental policies to mitigate ZIKV-related risks.

## Author summary

When cities lack proper planning, land use becomes disorganized, which creates ideal conditions for mosquitoes to thrive and spread diseases — especially affecting poorer communities. This study looked at how social and economic factors are connected to Zika virus infections in pregnant women and cases of microcephaly in babies in Recife, Brazil, between 2015 and 2021. The results showed that areas with higher poverty had more cases of Zika in pregnant women and more babies born with microcephaly. This highlights the strong link between social inequality and health problems. Both during and after the Zika outbreak, these issues continued to affect the most vulnerable neighborhoods in the city. These findings show how important it is to invest in public policies that improve living conditions and protect people socially and environmentally. Such actions are key to reducing the risk of Zika and other similar diseases, especially in the communities that need the most support.

## Introduction

Zika virus (ZIKV) is an arthropod-borne virus (arbovirus) belonging to the *Orthoflavivirus* genus of the *Flaviviridae* family [1,2]. In urban settings, ZIKV is primarily transmitted by the bite of *Aedes aegypti* mosquitoes, which are also vectors for dengue, chikungunya, yellow fever, and other viruses [3,4]. ZIKV infections during pregnancy can have adverse consequences for fetal and postnatal development and have, therefore, become a cause for concern, particularly for families planning to conceive and individuals who are pregnant [5,6].

After the emergence of an epidemic of an exanthematous disease in Brazil [7] which was confirmed in April 2015 to be the beginning of an explosive outbreak of ZIKV [8] and the subsequent detection of a cluster of microcephaly cases in October 2015 [9], national and international health authorities escalated operational surveillance measures, particularly focusing on ZIKV infections among pregnant individuals and microcephaly among newborns [10,11]. Although microcephaly (i.e., an abnormally small head circumference) can be caused by various factors (e.g., genetics, harmful substances, malnutrition) or have undefined etiology (i.e., idiopathic) [12], robust evidence has now confirmed the association between ZIKV infection during pregnancy and the development of microcephaly [13]. In addition, prenatal exposure to ZIKV has been associated with a range of outcomes including central nervous system damage including brain calcifications and ventriculomegaly, ocular anomalies, deafness, arthrogryposis, dysphagia, epilepsy, and delays in neurodevelopment – collectively recognized as Congenital Zika Syndrome (CZS) [3,6,14].

In many Brazilian cities, urbanization and population growth have resulted in sprawling, unplanned expansions of urban areas often lacking basic infrastructure, including adequate sanitation, access to clean drinking water, and environmental management [15]. This lack of structural planning leads to a chaotic, irregular occupation of space, which fosters the proliferation of mosquitoes and spread of infectious disease, with disproportionate impacts for the poorest segment of the population. Previous studies from the Brazilian cities of Rio de Janeiro [16–18] and Goiânia [19] have provided evidence of a higher frequency of ZIKV infections and CZS in more socioeconomically deprived areas. Similarly, a 2022 systematic review provided evidence of an association between indicators of lower socioeconomic position (e.g., lower education) and arboviral infection risks [20].

The hypothesis of the present study is that the distribution of ZIKV cases in pregnant women and microcephaly in newborns in the city of Recife is not homogeneous and is determined by the socioeconomic conditions of the environment. Using electronic health records collected between 2015–2021 in the city of Recife in the northeastern state of Pernambuco, which registered the highest number of cases in Brazil for 2016 [10,21], this study aims to understand the spatial distribution of ZIKV infections during pregnancy and live births with microcephaly and their associations with socioeconomic factors measured at the ecological level. This study will highlight spatial disparities in risk and identify census tracts that should be targeted for interventions to control mosquito vectors and reduce ZIKV transmission.

## Methods

### Ethics statement

The research was approved by the Ethics Committee of the HUOC/PROCAPE Hospital Complex, under CAAE No. 63869322.5.0000.5192 with approval report No. 5,738,832. This study did not require individual consent, as it was a study using secondary databases made available by the Pernambuco State Department of Health and the Recife Municipal Health Department, and followed the ethical procedures recommended for this type of study by Resolution MS/CNS 466/2012.

### Study design and data sources

This ecological study was conducted in Recife, the state capital of Pernambuco, Brazil, using the census tract as the spatial unit of analysis. The municipality of Recife is divided into 8 Health Districts, 94 official neighborhoods, and 1854 census tracts (based on the 2010 census). The population of Recife in 2022 (i.e., the time of the most recently available census data) was reported to be 1,488,920 inhabitants distributed across an area of 218,843 km^2^, resulting in a demographic density of 6,803.6 inhabitants/km^2^ [22].

This study analyzed administrative data collected between April 2015 and December 2021 from: (i) all pregnant persons registered as ZIKV-positive in the Brazilian Information System for Notifiable Diseases (*Sistema de Informação de Agravos de Notificaçao*, SINAN-Zika). ZIKV-positive in pregnant persons was confirmed when they presented pruritic maculopapular rash accompanied by two or more of the following signs and symptoms: fever, conjunctival hyperemia without secretion and pruritus, polyarthralgia, and periarticular edema. A suspected case was considered for ZIKV diagnosis if it tested positive on one of the following specific diagnostic methods: viral isolation, detection of viral RNA by reverse transcription polymerase chain reaction (RT-PCR), or IgM serology [23]. (ii) All live births diagnosed with microcephaly due to congenital ZIKV infection registered on the Public Health Event Registration System (*Registro de Eventos em Saúde Pública,* RESP-Microcefalia) and the Live Birth Information System (*Sistema de Informações Sobre Nascidos Vivos,* SINASC). Microcephaly cases were classified based on imaging and laboratory evidence. Cases were confirmed by imaging alone if the newborn presents with radiological findings suggestive of congenital infection, even in the absence of laboratory results. Laboratory-confirmed cases include those with microcephaly and a definitive diagnosis of either TORCH infections (syphilis, toxoplasmosis, rubella, cytomegalovirus, or herpes simplex) or Zika virus (ZIKV), based on conclusive laboratory testing of maternal and/or neonatal samples. In addition, microcephaly cases were confirmed when a newborn’s head circumference was ≤ 2 standard deviations below the mean for sex and gestational age according to the INTERGROWTH-21st growth curve used by the World Health Organization (WHO) and the Brazilian Ministry of Health [13,23]. Cases of microcephaly among stillbirths were not included in this study, as they are not recorded in SINASC. In addition, during the study period, only two stillbirths were reported in the Brazilian Mortality System (Sistema de Informações sobre Mortalidade, SIM), and their residential addresses were not registered in the system.

### Database linkage

First, both databases were standardized by removing duplicate entries. Subsequently, a probabilistic record linkage method was applied using a multi-step routine, where each step employed a specific blocking key. The next stage involved linking the datasets through the blocking process and record matching.

As comparison fields, the newborn’s full name and age were used, with linkage parameters estimated through the application of Expectation-Maximization (EM) algorithms. These parameters were then employed to calculate scores for the links established in each blocking step.

Finally, a single final dataset was generated, containing the true pairs identified based on the linkage score and manual review. The software Reclink III (version 3.1.6) and Microsoft Excel were used for this process.

### Georeferencing and variables

Cases of ZIKV infection in pregnant persons and microcephaly in live births were georeferenced using the residential address contained in the SINAN-Zika and RESP-Microcephalia/SINASC databases respectively, using the automatic tool MMQGIS/Geocode, which provides the geographic latitude and longitude coordinates of each address. The Google Maps/Open Street Map database was used as a reference integrated into QGIS 3.34. Subsequently, the specific georeferenced data were added to the census tract grid, thus preserving the location of the individual’s address.

Cases that could not be automatically located using the MMQGIS/Geocode tool were manually georeferenced, which involved searching for each address on the Recife City Hall spatial database and the postal system’s website. The digital cartographic base of the census tracts in the city of Recife were provided by the Brazilian Institute of Geography and Statistics (*Instituto Brasileiro de Geografia e Estatistica,* IBGE) website (www.ibge.gov.br) in shapefile format, available to free download in https://www.ibge.gov.br/geociencias/downloads-geociencias.html.

The primary study outcomes (i.e., the dependent variables) were the mean annual incidences of ZIKV infections among pregnant persons and microcephaly among live births. The mean annual incidence of ZIKV infections among pregnant persons was calculated, for each year, as the number of ZIKV cases in pregnant persons divided by the population of live births (as a proxy for the number of pregnant individuals) in the census tract and multiplied by one thousand pregnant persons. The mean annual incidence of microcephaly was calculated, for each year, as the number of cases of microcephaly divided by the population of live births in the census tract, multiplied by a thousand live births. Since there was a possible random fluctuation of the outcomes, the incidences were smoothed using the local empirical Bayes method [24,25]. To compare the spatial distribution of the study outcomes longitudinally, thematic maps were generated within two-time frames: from 2015 to 2017 during the epidemic period in Brazil and from 2018 to 2021 during the post-epidemic period in Brazil. All data were analyzed using QGIS 3.26 and GeoDa 1.8.

The exposure (i.e., independent) variables investigated social, economic, sanitation and urban infrastructure at the census tract level as recorded in the 2010 IBGE database. Specifically, these variables included: (i) the proportion of households with a nominal monthly household income per capita ≤1 minimum wage (i.e., 510 BRL, approximately 290 USD in 2010); (ii) the proportion of households with a water supply other than that supplied via the main distribution network (i.e., via a well or spring, rainwater stored in cisterns, or other sources); (iii) the proportion of households linked to a sewage system via the main sewage or rainwater network; (iv) the proportion of households with no bathroom; (v) the proportion of households with inappropriate waste disposal (i.e., burned or buried on the property, thrown onto vacant land or street, thrown into a river, lake or sea or other destinations); (vi) the proportion of households with inappropriate sewage disposal; (vii) the proportion of households with accumulated garbage on the streets; (viii) the proportion of households with electricity; (ix) the proportion of racially minoritized (i.e., *Preta* [Black] and *Parda* [Brown/mixed race]) women aged >10 years; and (x) the proportion of illiterate women aged over 10 years.

### Spatial analysis

For the spatial analysis, the bivariate global Moran’s I was first calculated between the incidences of ZIKV infections among pregnant persons and microcephaly among live births during the two time periods (i.e., 2015–2017 and 2018–2021). It tests the spatial dependence among observations. I > 0 indicates direct correlation (clustering) between neighbors. When I < 0, indicate negative or inverse correlations (i.e., dispersion). And when I = 0, there is no spatial autocorrelation [24]. Positive spatial autocorrelation indicates that areas with higher proportions of the independent variable (i.e., socioeconomic indicator) tend to cluster with higher incidences of the outcome (i.e., ZIKV infection in pregnant persons or microcephaly), while negative autocorrelation suggests an inverse relationship.

To visualize the spatial distribution of census tracts with clusters of risks of ZIKV infections in pregnant persons and microcephaly, LISA Maps were generated for each of the time periods. This allows the detection of census tracts with spatial dependencies that are not illustrated through global indices. Two types of maps were used in the Moran autocorrelation test: i) a BoxMap map showing clusters with high–high patterns (i.e., areas where a specific census tract and its neighbors show high values), low-low patterns (i.e., areas where a specific census tract and its neighbors show low values), low-high patterns (i.e., areas where a specific census tract has a low value and its neighbors have a high value), and high-low patterns (i.e., areas where a specific census tract has a high value and its neighbors have a low value); (ii) a MoranMap map showing statistically significant high-high, low-low, low-high, and high-low clusters; indicating the magnitude of the p-value for each census tract, with statistical significance defined according to a two-sided threshold of p < 0.05 [24].

## Results

### Sociodemographic characteristics

From 2015 to 2021, the city of Recife registered 253 cases (n = 240 in 2015–2017 and n = 13 in 2018–2021) of ZIKV infection in pregnant persons and 147 cases (n = 137 in 2015–2017 and n = 10 in 2018–2021) of microcephaly in newborns. The registered cases of pregnant persons infected with ZIKV infection were primarily aged between 21 and 30 years (43.9%, n = 111/253), identified as *Parda* (Brown) (71.2%, n = 131/184), and had between 10–12 years of schooling (55.5%, n = 101/182) (Table 1).

**Table 1:** Sociodemographic characteristics of the registered cases of pregnant persons infected with ZIKV in Recife (2015-2021).

Characteristics	Number (%)
**Number of pregnant persons included in the study**	253
**Age group**	
≤20 years	63 (24.9%)
21 to 30 years	111 (43.9%)
31 to 40 years	60 (23.7%)
>40 years	19 (7.5%)
**Race/ethnicity**	
*Branca,* white	38 (20.7%)
*Parda,* Brown	131 (71.2%)
*Preta,* Black	15 (8.1%)
Missing	69
**Educational level (years of study)**	
≤4 years	2 (1.1%)
5 to 9 years	66 (36.3%)
10 to 12 years	101 (55.5%)
>12 years	13 (7.1%)

Among the 147 cases of microcephaly, these children were most frequently born to mothers who were aged between 21 and 30 years (49.3%, n = 71/143) and identified as *Parda* (Brown) (73.3%, n = 55/75). The majority of registered microcephalic newborns with available data were born at full-term (i.e., ≥ 37-weeks of gestational age; 86.3%, n = 120/139), were female (51.7%, n = 75/145), and had birth weight ≥ 2500g (80.1%, n = 113/141). The median (interquartile range) for birth length was 46 (45–47) cm (Table 2).

**Table 2:** Sociodemographic and birth characteristics of the registered cases of live births with ZIKV-related microcephaly in Recife (2015-2021).

Characteristics	Number (%)
**Number included in the study**	**147 newborns**
**Maternal age**	
≤20 years	26 (18.0%)
21 to 30 years	71 (49.3%)
31 to 40 years	40 (27.8%)
>40 years	7 (4.9%)
Missing	3
**Maternal race/ethnicity**	
*Branca,* white	11 (14.7%)
*Parda,* Brown	55 (73.3%)
*Preta,* Black	9 (12.0%)
Missing	72
**Gestational age**	
Term	120 (86.3%)
Preterm	19 (13.7%)
Missing	8
**Sex**	
Male	70 (48.3%)
Female	75 (51.7%)
Missing	2
**Birth weight**	
≥2500g	113 (80.1%)
<2500g	28 (19.9%)
Missing	6
**Length (in cm)**	
≤ 43 cm	3 (5.4%)
44 cm to 45 cm	19 (33.9%)
46 cm to 47 cm	22 (39.3%)
48 cm to 49 cm	9 (16.0%)
≥ 50 cm	3 (5.4%)
Missing	91

### Bivariate spatial dependence of the incidence of ZIKV infection in pregnant persons and microcephaly

Analyzing the bivariate spatial dependence between the incidences of ZIKV infections in pregnant persons and microcephaly, the value of the global Moran’s I index were 0.127 (p = 0.001) for the period between 2015 and 2017 and 0.074 (p = 0.002) for the period between 2018 and 2021, thereby indicating the presence of spatial risk clusters in the two periods analyzed.

**Fig 1:**
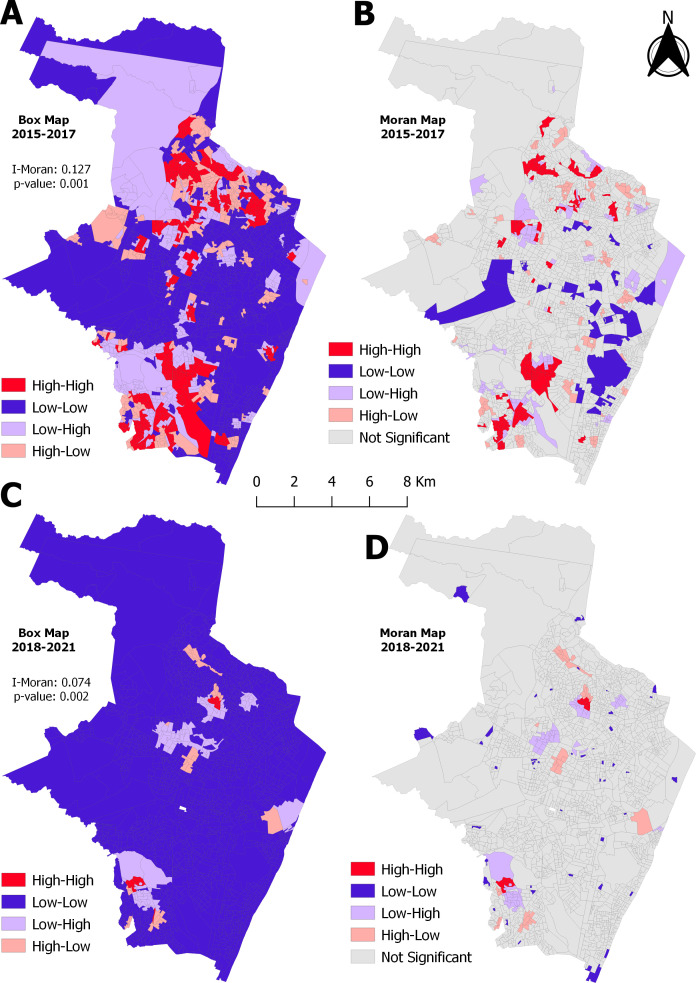
Bivariate spatial dependence (Box Map and Moran Map) of the incidence of ZIKV in pregnant persons with microcephaly. Clusters areas in red (High-High) represent the region with the highest incidence of ZIKV infection in pregnant persons and higher incidence of microcephaly in each map: (Map A and B) period 2015-2017 and (Map C and D) period 2018-2021. The cartographic base that served as the basis for the production of the maps is at https://www.ibge.gov.br/geociencias/downloads-geociencias.html.

During the epidemic period from 2015 to 2017, 16.7% (n = 309/1854) of the census tracts were classified as both high risk for ZIKV infection in pregnant persons and high risk for microcephaly, while 53.0% (n = 983/1854) of the tracts were classified as low risk in both measures (Fig 1A). Considering the hypothesis of local spatial correlation for each census tracts, (Fig 1B), 19% of the census tracts presented a statistical significance (i.e., I ≠ 0 and p-value < 0.05). During the post-epidemic period from 2018 to 2021, the incidence of both measures decreased, and only 0.9% (n = 16/1854) of the census tracts were classified as both high risk for ZIKV infection in pregnant persons and high risk for microcephaly, while 91.8% of the tracts (n = 1702/1854) were classified as low risk in both measures.

Considering the hypothesis of local spatial correlation for each census tracts, (Fig 1C), 9% of the census tracts presented a statistical significance (i.e., I ≠ 0 and p-value < 0.05).

By taking into account only the tracts that were high-risk for both ZIKV infections during pregnancy and microcephaly, high-risk clusters covering 119 tracts were identified in the north, central-west and south zones during the period from 2015 to 2017 (Fig 1B). During the period from 2018 to 2021, 14 tracts were considered high-risk clusters (Fig 1D).

### Bivariate spatial correlation between the incidences of ZIKV infection in pregnant persons and microcephaly with socioeconomic indicators

According to the values of the global bivariate Moran’s I in Table 3, the incidence of both ZIKV infection in pregnant persons and microcephaly exhibited significant spatial autocorrelation for most variables studied across both analyzed periods. For example, positive spatial autocorrelation was observed between the proportion of households with a nominal monthly household income per capita ≤1 minimum wage and incidence of microcephaly (i.e., the higher the proportion of households with a nominal monthly household income per capita ≤1 minimum wage, the higher the incidence of microcephaly). While negative autocorrelation was observed between the the proportion of households linked to a sewage system via the main sewage or rainwater network and incidence of microcephaly (i.e., the lower the proportion of households with a nominal monthly household income per capita ≤1 minimum wage, the higher the incidence of microcephaly).

**Table 3:** Bivariate Global Moran’s I between the incidences of ZIKV infection in pregnant persons and microcephaly with socioeconomic indicators measured at the census tract level.

Independent variables	Incidence of microcephaly (2015 – 2017)	p-value	Incidence of microcephaly (2018 – 2021)	p-value	Incidence of ZIKV in pregnant persons (2015 – 2017)	p-value	Incidence of ZIKV in pregnant persons (2018 – 2021)	p-value
Proportion of households with a nominal monthly household income per capita ≤1 minimum wage (i.e., 510 BRL, approximately 290 USD in 2010)	0.107	**0.001**	0.052	**0.001**	0.180	**0.001**	0.084	**0.001**
Proportion of households with a water supply other than that supplied via the main distribution network (i.e., via a well or spring, rainwater stored in cisterns, or other sources)	0.001	0.433	-0.019	**0.020**	0.001	0.422	-0.008	0.212
Proportion of households linked to a sewage system via the main sewage or rainwater network	-0.094	**0.001**	0.008	0.205	-0.182	**0.001**	-0.097	**0.001**
Proportion of households with no bathroom	0.002	0.381	0.017	0.067	0.038	**0.009**	0.034	**0.009**
Proportion of households with inappropriate waste disposal (i.e., burned or buried on the property, thrown onto vacant land or street, thrown into a river, lake or sea or other destinations)	0.012	0.128	0.021	**0.039**	0.016	0.081	0.025	**0.019**
Proportion of households with inappropriate sewage disposal	0.119	**0.001**	0.020	**0.041**	0.004	0.371	0.020	**0.033**
Proportion of households with accumulated garbage on the streets	0.015	0.077	-0.021	**0.013**	-0.042	**0.001**	-0.023	**0.001**
Proportion of households with electricity	-0.049	**0.001**	0.000	0.398	-0.030	**0.013**	0.006	0.296
Proportion of racially minoritized (i.e., Preta [Black] and Parda [Brown/mixed race]) women aged >10 years	0.097	**0.001**	0.048	**0.001**	0.158	**0.001**	0.076	**0.001**
Proportion of illiterate women aged over 10 years	0.050	**0.001**	0.022	**0.023**	0.109	**0.001**	0.053	**0.001**

### Spatial correlation between ZIKV incidence in pregnancy and socioeconomic indicators

**Fig 2:**
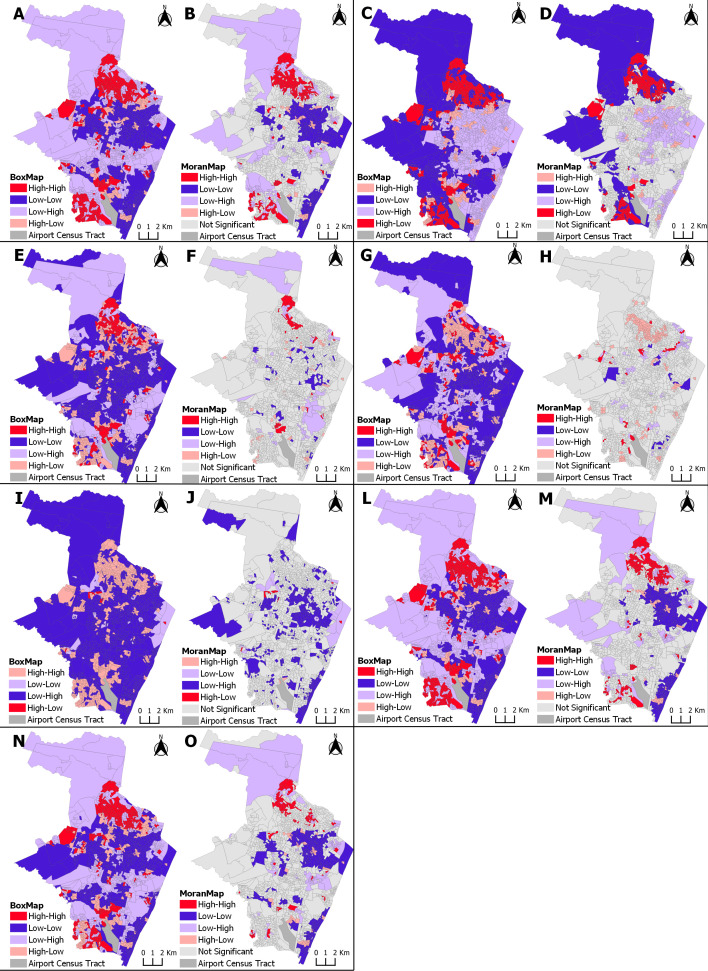
Spatial correlation between ZIKV incidence in pregnancy and socioeconomic indicators, showing high- and low-risk clusters. Recife, Pernambuco, 2015-2017. Clusters areas in red represent the region with highest incidence of ZIKV and higher vulnerability by socioeconomic variables in each map: (Map A and B) Proportion of households with a nominal monthly household income per capita of up to 1 minimum wage. (Map C and D) Proportion of households linked to a sewage system via the main sewage or rainwater network. (Map E and F) Proportion of households with no bathroom. (Map G and H) Proportion of households with accumulated garbage in public places. (Map I and J) Proportion of households with electricity. (Map L and M) Proportion of Black color/race (black and brown) women aged over 10 years. (Map N and O) Proportion of women aged over 10 years with low literacy skills. The cartographic base that served as the basis for the production of the maps is at https://www.ibge.gov.br/geociencias/downloads-geociencias.html.

By analyzing the Box Map and Moran Map with the bivariate spatial dependence between the incidence of ZIKV infection in pregnant persons and the covariates for the periods analyzed, the most vulnerable census tracts were highlighted (Fig 2). Among the independent variables that presented a positive correlation with the incidence of ZIKV infection in pregnant persons, the High-High standard tracts are classified as the areas of greatest vulnerability (i.e., low socioeconomic level) in relation to the risk of infection (high incidence of ZIKV in pregnant persons with a high proportion of the explanatory socioeconomic indicator variable). Among the variables that presented a negative autocorrelation with the event studied, the tracts that presented a High-Low pattern are the areas of greatest vulnerability (i.e., low socioeconomic level) in relation to the risk of infection, in this case, a high incidence of ZIKV in pregnant persons with a low proportion of the explanatory variable.

**Fig 3:**
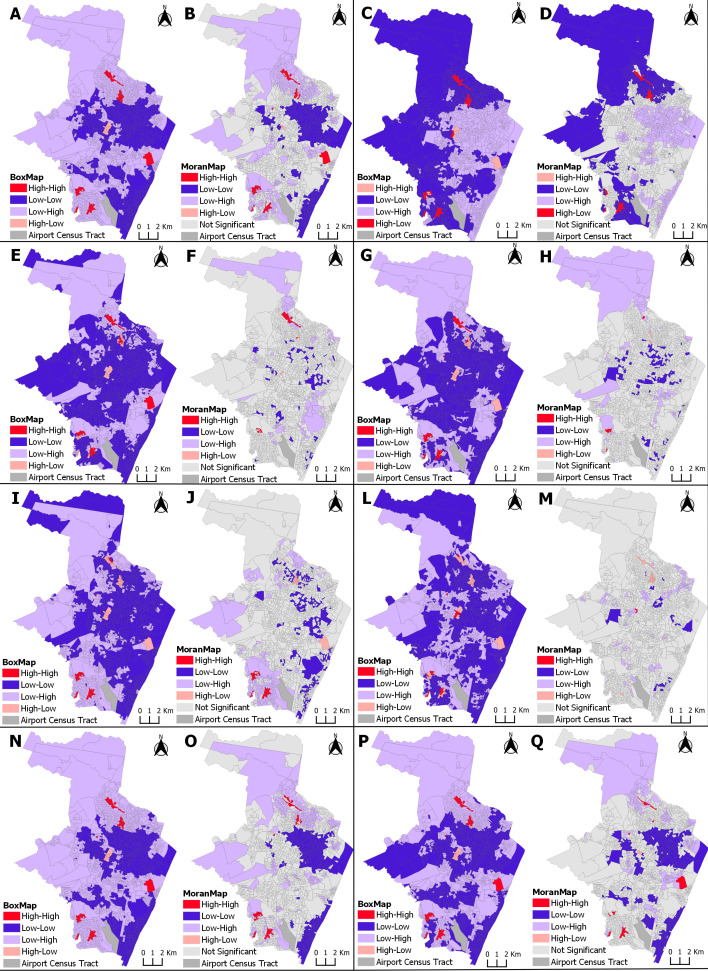
Spatial correlation between ZIKV incidence in pregnancy and socioeconomic indicators, showing high- and low-risk clusters. Recife, Pernambuco, 2018-2021. Clusters areas in red represent the region with the highest incidence of ZIKV and higher vulnerability by socioeconomic variables in each map: (Map A and B) Proportion of households with a nominal monthly household income per capita of up to 1 minimum wage. (Map C and D) Proportion of households linked to a sewage system via the main sewage or rainwater network. (Map E and F) Proportion of households with no bathroom. (Map G and H) Proportion of households with inappropriate waste disposal. (Map I and J) Proportion of households with an open sewage system in public areas. (Map L and M) Proportion of households with accumulated garbage in public places. (Map N and O) Proportion of Black color/race (black and brown) women aged over 10 years. (Map P and Q) Proportion of women aged over 10 years with low literacy skills. The cartographic base that served as the basis for the production of the maps is at https://www.ibge.gov.br/geociencias/downloads-geociencias.html.

Fig 2 presents the maps with the risk zones for ZIKV infection in pregnant persons, for each of the explanatory socioeconomic indicator variables, from 2015 to 2017, and Fig 3 from 2018 to 2021.

### Spatial correlation between the incidence of microcephaly and socioeconomic indicators

**Fig 4:**
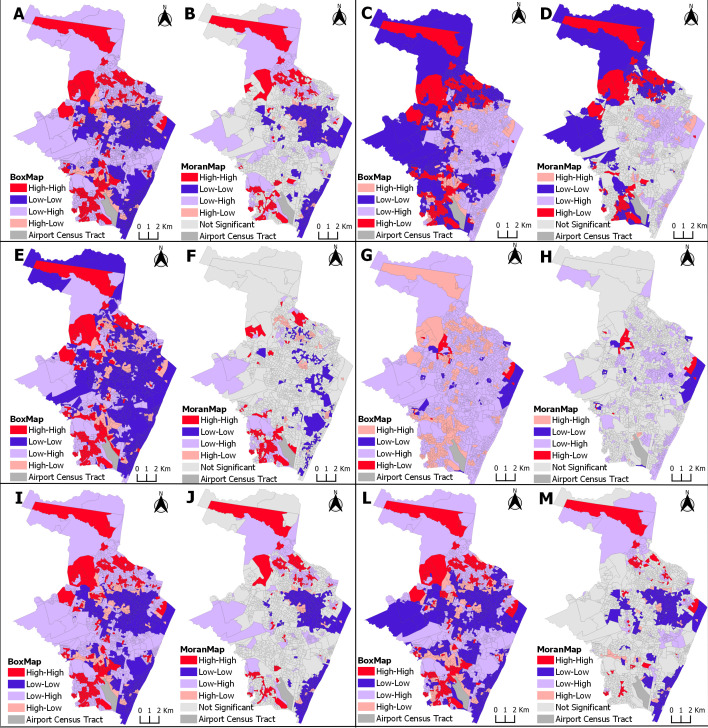
Spatial correlation between the incidence of microcephaly and socioeconomic indicators, showing high- and low-risk clusters. Recife, Pernambuco, 2015-2017. Clusters areas in red represent the region with the highest incidence of microcephaly and higher vulnerability by socioeconomic variables in each map: (Map A and B) Proportion of households with a nominal monthly household income per capita of up to 1 minimum wage. (Map C and D) Proportion of households linked to a sewage system via the main sewage or rainwater network. (Map E and F) Proportion of households with an open sewage system in public areas. (Map G and H) Proportion of households with electricity. (Map I and J) Proportion of Black color/race (black and brown) women aged over 10 years. (Map L and M) Proportion of women aged over 10 years with low literacy skills. The cartographic base that served as the basis for the production of the maps is at https://www.ibge.gov.br/geociencias/downloads-geociencias.html.

**Fig 5:**
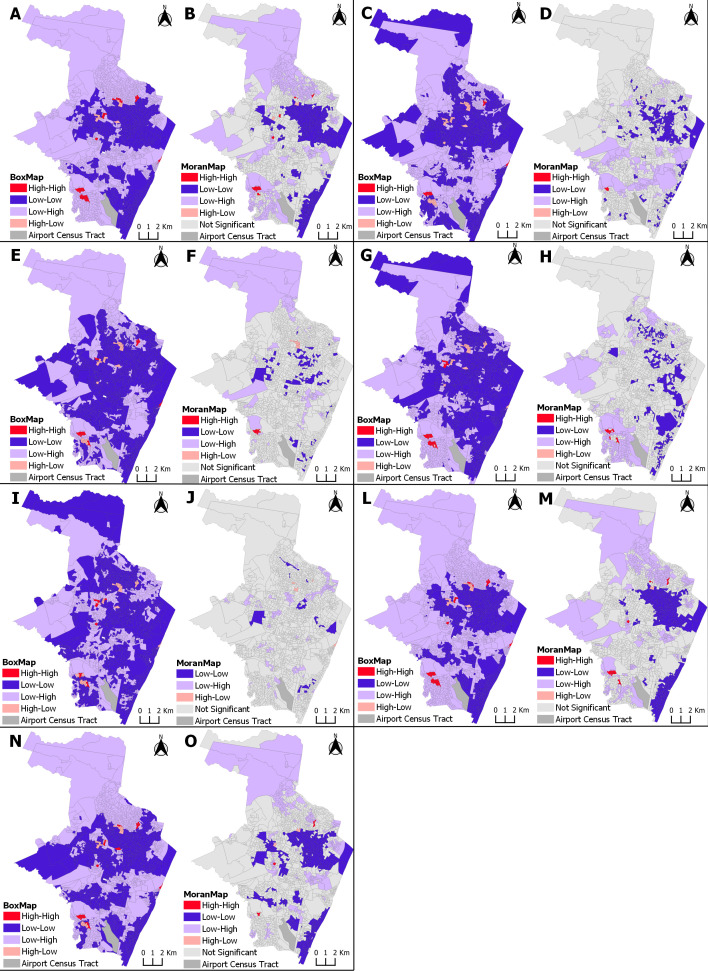
Spatial correlation between the incidence of microcephaly and socioeconomic indicators, showing high- and low-risk clusters. Recife, Pernambuco, 2018-2021. Clusters areas in red represent the region with the highest incidence of microcephaly and higher vulnerability by socioeconomic variables in each map: (Map A and B) Proportion of households with a nominal monthly household income per capita of up to 1 minimum wage. (Map C and D) Proportion of households with a water supply other than that supplied via the main distribution network. (Map E and F) Proportion of households with inappropriate waste disposal. (Map G and H) Proportion of households with an open sewage system in public areas. (Map I and J) Proportion of households with accumulated garbage in public places. (Map L and M) Proportion of Black color/race (black and brown) women aged over 10 years. (Map N and O) Proportion of women aged over 10 years with low literacy skills. The cartographic base that served as the basis for the production of the maps is at https://www.ibge.gov.br/geociencias/downloads-geociencias.html.

In Figs 4 and 5, the maps describe the risk zones of microcephaly, for each of the explanatory variables, in each of the periods. The maps indicate the tracts of greatest vulnerability in relation to the risk of a child living in that tract and developing microcephaly, given that the census tract has a high incidence of microcephaly with a high proportion of the explanatory variable. For example, the maps indicate a greater risk of microcephaly in tracts with a higher proportion of households with a nominal monthly household income per capita ≤1 minimum wage.

## Discussion

Recife emerged as the epicenter of the ZIKV epidemic in Brazil, registering 253 confirmed cases of ZIKV infection among pregnant persons and 147 confirmed cases of microcephaly in newborns between 2015 and 2021. This ecological study has revealed a geographic pattern of spatial distribution, with confirmed cases of ZIKV in pregnant persons and microcephaly clustering in the north, central-west, southeast, and south zones of the municipality based on census tract data. Furthermore, this study has demonstrated that incidences of maternal ZIKV infection and microcephaly were consistently associated with indicators of greater social vulnerability and economic deprivation at the census tract level. It should be noted that the Moran’s index correlations remained consistent across the two analyzed periods, even with reduced ZIKV circulation in the post-epidemic period.

Our findings indicate the presence of spatial risk clusters, with an overlap in the areas experiencing increased incidences of maternal ZIKV infections and microcephaly. High-risk areas for ZIKV outcomes were concentrated in ‘pockets of poverty,’ even within wealthier neighborhoods. Our data indicate elevated risks in census tracts with inadequate infrastructure characterized by a higher proportion of households experiencing a lack of basic sanitation with the presence of open sewage, inadequate waste management, and/or a lack of electricity. In addition, incidences of maternal ZIKV infections and microcephaly were also found to correlate with social determinants of health, with clusters found in communities with high proportions of households with low per capita income, of racially minoritized women, and/or of women with low literacy.

In line with the findings of this research, two previous studies carried out in Recife confirmed a strong correlation between the distribution of maternal ZIKV infections [26] and microcephaly cases with poverty [27]. Notably, our results contrast the findings of a study carried out in the state of Rio Grande do Norte, Brazil, in which the highest notification of cases due to ZIKV infection was found in municipalities with a higher level of income, such as the state capital, Natal; the authors suggested the increased notifications in these areas likely reflect increased knowledge regarding the disease, better public health infrastructure, and a greater access to health services [28].

Although registered rates of ZIKV infection and cases of ZIKV-related microcephaly currently remain low, new cases continue to be reported across the country of Brazil with low seasonal peaks during the dry season between May and October [15]. The present study highlights geographic regions of Recife with heightened risks for the emergence of a new epidemic of ZIKV. Our findings demonstrate that, even after the epidemic period, cases of ZIKV in pregnant persons and microcephaly were concentrated in census tracts with relatively higher socioeconomic vulnerability, reinforcing the need for research to inform the development of social protection and environmental policies to mitigate ZIKV-related risks within these communities. Specifically, public policies should aim to address social inequalities and be tailored to effectively support populations with low purchasing power and those facing barriers to accessing high-quality health and education services.

Moreover, the infrastructure deficits of the high-risk areas (e.g., open sewage and inadequate waste disposal) present a favorable environment for breeding of *Aedes spp.* Mosquitoes [29]. Accelerated, unplanned urbanization processes contribute to socio-spatial weaknesses and an ecological imbalance, facilitating the proliferation of the *Aedes spp.* mosquitoes, thereby expanding the geographic range of arbovirus infections linked to the urban environment [28]. This study highlights the need for more effective actions and planning for environmental and vector control policies to combat the proliferation of *Aedes* mosquitoes, particularly within these high-risk clusters experiencing weaknesses in basic infrastructure [30–32].

The strengths and limitations of this study warrant consideration. This study has leveraged spatial autocorrelation in order to analyze the census tracts most vulnerable to ZIKV infection among pregnant persons and to microcephaly cases. The analysis has revealed disparities in risks between census tracts and also highlighted the influence of neighboring census tracts. While the global incidence serves as a good population-level disease indicator, local Moran’s I spatial statistical correlation provides a more refined understanding of ZIKV infection and microcephaly, directly linking them to socioeconomic factors and social determinants measured at the census tract level. Another strength is that this study used census tracts, which allowed for a more precise spatial visualization of the areas where the highest levels of incidence were concentrated. The chief limitations of the study involved the potential underreporting of cases. However, the surveillance of ZIKV did not change overtime therefore we would not expect that underreporting contributed significantly to this decrease. In addition, if underreporting occurred it is not likely that it was differential in areas with different socioeconomic conditions as notification of gestational infection and microcephaly were made compulsory by the Ministry of Health and the Zika laboratory tests were only available in the public sector. Therefore, it could impact in the number of cases, but not the spatial association between infection and socioeconomic conditions. Notably, a high proportion of ZIKV infections will be asymptomatic [33] or present with only mild symptoms and may, therefore, never registered in the SINAN-Zika notification system. Among cases submitted to the registration system, a subset of cases may remain unconfirmed while they remain under investigation. The use of secondary data may also introduce inconsistencies regarding the quality of the data provided, such as the high frequency of missing data, particularly regarding maternal race/ethnicity and educational level. It should also be highlighted that in the post-epidemic period, there was the COVID-19 pandemic, which may have interfered with notifying cases of maternal ZIKV infection and microcephaly. It is possible that uncontrolled variables may have influenced spatial correlations. As we used secondary data from the official dataset (RESP, SINAN, SINASC), it was not possible to adjust to confounders as this information was not available. However, Recife has an area of only 218.84 Km^2^, the climate is the same for the whole area. The climate may have changed from the epidemic period to the pos epidemic period. It would influence the number of cases, but we would not expect that it would affect the spatial correlation between socioeconomic conditions and ZIKV infection. In relation to coinfection, primary data from some studies showed that women were coinfected so the other infection (Chikungunya, Dengue and TORCH) [5] would not affect the spatial distribution of ZIKV infection.

Preventing and combating a new ZIKV epidemic will be no simple task. Overall, our findings provide robust evidence that the risks of ZIKV infections in pregnancy and microcephaly in Recife were increased in census tracts with increased deprivation and lower socioeconomic positions. More broadly, our findings reinforce the importance of risk mapping to inform effective planning to prevent infectious disease epidemics. The spatial distribution of socioeconomic inequalities should be taken into account when organizing strategic public health interventions, such as vector control activities, targeting the provision of social assistance, improving sanitation, social protection policies and continuous epidemiological monitoring, prioritizing areas with higher vulnerability. In the future, we recommend the use of more sophisticated spatial statistics tools that take into account the interrelation between the explanatory variables and their correlation with ZIKV infection and microcephaly.

## Supporting information

S1 Table Supplementary table. Total percentages of each socioeconomic variables according 2010 Demographic Census (IBGE). Recife, Pernambuco.
(DOCX)
