# Supplementary material for: Mapping the risk of Zika virus infections in pregnant persons and microcephaly in newborns in relation to socioeconomic indicators in Recife, Pernambuco, Brazil: A spatial analysis (2015 to 2021)
Source: PLoS Negl Trop Dis. 2025 Jul 9;19(7):e0013240. doi: 10.1371/journal.pntd.0013240 (PMC12266408; doi:10.1371/journal.pntd.0013240)
Supplement: S1 Table — Total percentages of each socioeconomic variables according 2010 Demographic Census (IBGE). Recife, Pernambuco. (DOCX) [file pntd.0013240.s001.docx]

# Supplementary Table. Total percentages of each socioeconomic variables according 2010 Demographic Census (IBGE). Recife, Pernambuco.

| **Independent variables** | **Percentage** |
| --- | --- |
| Proportion of households with a nominal monthly household income per capita ≤1 minimum wage (i.e., 510 BRL, approximately 290 USD in 2010) | 54.6% |
| Proportion of households with a water supply other than that supplied via the main distribution network (i.e., via a well or spring, rainwater stored in cisterns, or other sources) | 7.2% |
| Proportion of households linked to a sewage system via the main sewage or rainwater network | 54.8% |
| Proportion of households with no bathroom | 0.5% |
| Proportion of households with inappropriate waste disposal (i.e., burned or buried on the property, thrown onto vacant land or street, thrown into a river, lake or sea or other destinations) | 2.2% |
| Proportion of households with inappropriate sewage disposal | 12.2% |
| Proportion of households with accumulated garbage on the streets | 4.2% |
| Proportion of households with electricity | 99.1% |
| Proportion of racially minoritized (i.e., *Preta* [Black] and *Parda* [Brown/mixed race]) women aged >10 years | 60.6% |
| Proportion of illiterate women aged over 10 years | 7.4% |
